# Obesity-induced Endothelial Dysfunction is Prevented by Neutrophil Extracellular Trap Inhibition

**DOI:** 10.1038/s41598-018-23256-y

**Published:** 2018-03-20

**Authors:** Hui Wang, Qian Wang, Jessica Venugopal, Jintao Wang, Kyle Kleiman, Chiao Guo, Daniel T. Eitzman

**Affiliations:** 1 University of Michigan, Department of Internal Medicine, Cardiovascular Research Center, Ann Arbor, Michigan USA; 2 Department of Cardiology, the Fourth Affiliated Hospital of China Medical University, Shenyang, China

## Abstract

Endothelial dysfunction precedes atherosclerosis and may constitute a critical link between obesity-related inflammation and cardiovascular disease. Neutrophil extracellular traps (NETs) have been shown to promote vascular damage in murine models of autoimmune disease and atherosclerosis. The impact of NETs towards endothelial dysfunction associated with obesity is unknown. Using a diet-induced obesity (DIO) mouse model, this study investigated whether the inhibition or degradation of NETs could reduce the endothelial dysfunction observed in DIO mice. Following induction of DIO, there were elevated plasma concentrations of monocyte chemoattractant protein-1 (MCP-1) and impairment of mesenteric arteriolar vasorelaxation in response to acetylcholine as measured by pressure myography. A marker of NET formation, cathelicidin-related antimicrobial peptide (CRAMP), was markedly increased in mesenteric arterial walls of DIO mice compared to mice on standard chow. Prevention of NET formation with Cl-amidine or dissolution of NETs with DNase restored endothelium-dependent vasodilation to the mesenteric arteries of DIO mice. These findings suggest an instrumental role for NETs in obesity-induced endothelial dysfunction.

## Introduction

Approximately two thirds of Americans are overweight or obese^1^. Increased adiposity is associated with a chronic inflammation^2^ which may contribute to cardiovascular morbidity and mortality. Recent studies have revealed that mice fed a high-fat diet are more prone to spontaneous neutrophil extracellular trap (NET) formation^3–5^. NETs are proinflammatory, microbial web-like structures of chromatin, entangled with histones and other proteins^6^. NETs were first shown to extrude from neutrophils in response to certain stimuli, such as cytokines and microbial products^6^, however other upstream triggers of NET formation remain to be elucidated. The presence of NETs can be specifically inhibited by Cl-amidine^7,8^, an inhibitor of peptidylarginine deiminase 4 (PAD4), whose action is necessary for histone citrinullation during NET formation^9–11^. DNase treatment can degrade NETs and thereby negate their action^12^.

NET formation has also been shown to negatively impact aspects of endothelial function^13–16^ and to promote vascular damage induced in an apolipoprotein E deficient model of atherosclerosis^17^. The atherosclerosis in this model was ameliorated by NET inhibition.

The current study was designed to determine whether targeting formation or removal of NETs in a model of DIO could prevent endothelial dysfunction. These findings suggest an instrumental role for NETs in obesity-induced vascular pathologies.

## Methods

### Animals

Male C57BL6/J mice were purchased from Jackson Laboratory (Bar Harbor, Maine). Mice were fed either a standard laboratory rodent diet (No. 5001, TestDiet, Richmond, IN) or a high fat, high sucrose diet (HFD) (D12451, Research Diet Inc, New Brunswick, NJ) and tap water ad libitum in a temperature-controlled room with a 12:12-hour light/dark cycle. HFD was given for 10 weeks, beginning at 8 weeks of age. All animal use protocols complied with the Principle of Laboratory and Animal Care established by the National Society for Medical Research and were approved by the University of Michigan Committee on Use and Care of Animals.

### Neutrophil extracellular trap (NET) inhibition

A peptidylarginine deiminase inhibitor, Cl-amidine, was used to block NET formation. At 16 weeks of age, (8 weeks after initiation of high fat, high sucrose diet) DIO mice were treated with Cl-amidine dissolved in 200 μl PBS by daily subcutaneous injection (10 mg/kg/d) or 200 μl PBS control for 2 weeks. Vascular function of mesenteric arterioles was then examined using pressure myography as described previously^18^.

To further study the causal role of NETs on DIO-induced vascular dysfunction, deoxyribonuclease (DNase) (Genentech, South San Francisco, CA) was used to degrade NETs. 50 µg of DNase in a volume of 50uL was injected intraperitoneally daily for 8 days (9 weeks after initiation of the high fat, high sucrose diet) to DIO mice. This dose, up to 2×/day, has been used in models of thrombosis and shown to reduce thrombogenicity in certain disease states^19,20^. We have also previously used this dose in a lupus model of thrombosis^7^. A control vehicle was given in identical volume. Control vehicle was also given for mice treated with Cl-amidine. Vascular function of mesenteric arterioles was then examined using pressure myography (Table 1).

**Table 1:**
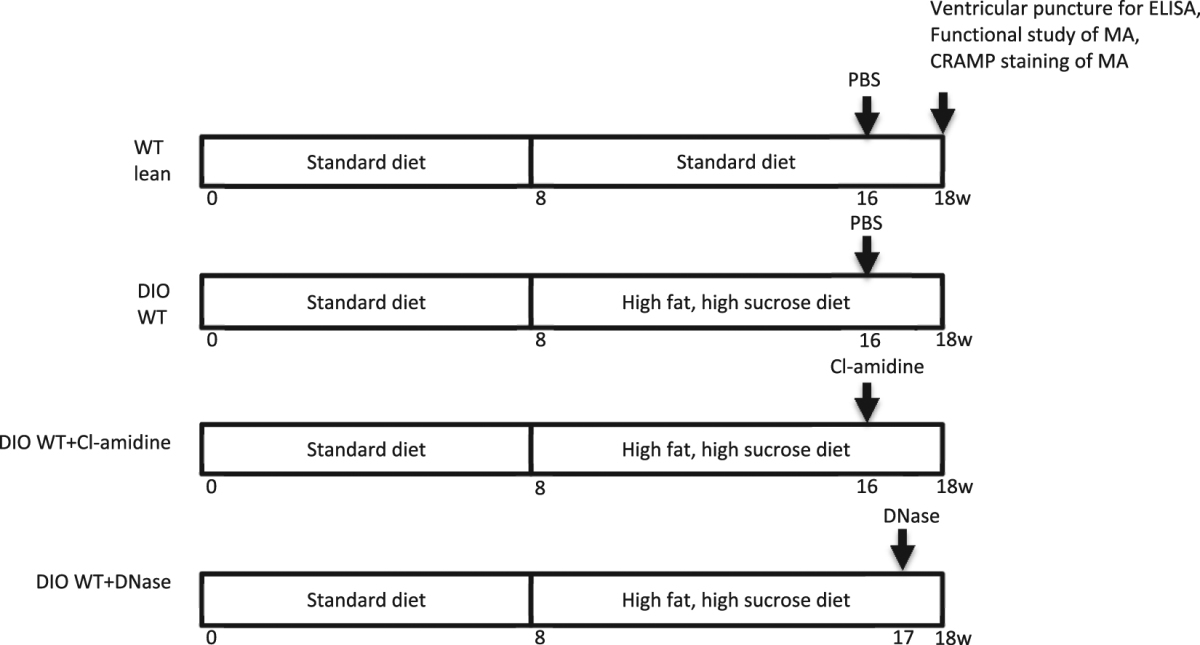
Overview of experimental plan.

### Immunohistochemistry

Mesenteric arteries were collected and fixed in zinc formalin as described previously^18^. NET content in paraffin-embedded mesenteric arterial sections was identified by staining of the NET-related marker cathelicidin-related antimicrobial peptide (CRAMP) using a rabbit anti-mouse CRAMP polyclonal antibody (1:200) (Innovagen, Lund, Sweden). Positive stained area was detected with a biotin-conjugated secondary antibody and analyzed by automatic detection of positive staining intensity using Nikon MetaMorph software. Positive staining area was expressed as a percentage of the total medial area of blood vessel wall.

### Measurement of plasma factors

Plasma samples were collected via ventricular puncture at the time of euthanasia. Plasma monocyte chemoattractant protein-1 (MCP-1), interleukin-6 (IL-6), vascular cell adhesion molecule 1 (VCAM-1), and fasting insulin levels were measured with commercially available murine ELISA kits (MCP-1, IL-6, VCAM-1: R&D Systems, Minneapolis, MN; insulin: Crystal Chemical Inc., Wakefield, MA) according to manufacturers’ instructions. Overnight fasting blood glucose levels were measured using an Ascensia Contour Blood Glucose Meter and Ascensia Contour test strips (Bayer Healthcare LLC, Tarrytown, NY).

### Functional studies of mesenteric arterioles

Functional studies of mesenteric arterioles were performed as previously described^18^. Briefly, mice were euthanized with intraperitoneal pentobarbital (80 mg/kg) and a segment of small intestine with attached mesentery was removed and placed into a silastic-elastomer lined petri dish filled with cold PSS equilibrated with 5% CO_2_–95% O_2_. The second-order branches of mesenteric arterioles were dissected, and mounted onto glass cannulae of a pressure myograph (Living Systems, VT). The real-time dimension of the vessel wall was detected and analyzed by a video dimension analyzer (Living Systems, VT). Vascular contraction was assessed by measuring constriction in response to cumulatively applied norepinephrine (NE, Sigma, St. Louis, MO) (10^−8^ to 10^−4^ mol/L). After washing and equilibration, endothelium-dependent relaxation was assessed by measuring the dilatory response to acetylcholine (Ach, Sigma) (10^−9^ to 10^−4^ mol/L) in NE precontracted vessels (10^−5^ mol/L). To evaluate NO bioavailability, Ach-induced vessel relaxation was assessed after vessels were incubated for 20 min with the NO synthase inhibitor Nω-nitro-l-arginine methyl ester (L-NAME, 10^−4^ mol/L). Endothelium-independent relaxation was assessed by extraluminally applied sodium nitroprusside (SNP, Sigma, St. Louis, MO) (10^–9^ to 10^–3^ mol/L) on the same vessel precontracted with NE (10^–5^ mol/L).

### Statistical analysis

All data are presented as mean ± standard error. Statistical analysis was carried out using GraphPad Prism. Results were analyzed using unpaired t-test for comparison between two groups. Tests for normality were performed using the Shapiro-Wilk test. For multiple comparisons, results were analyzed using one-way or two-way ANOVA followed by Turkey post-test analysis. Probability values of p < 0.05 were considered statistically significant.

## Results and Discussion

Following 10 weeks of a high fat, high sucrose diet, body weight and fasting blood glucose levels were elevated compared to mice on standard diet and they were not affected by treatment with either Cl-amidine or DNase (Table 2). As expected, fasting plasma insulin levels were also increased in DIO WT mice compared with control WT lean mice (Table 2). Thus targeting NET formation or degradation at 8 or 9 weeks after DIO initiation, respectively, does not appear to affect glucoregulation. This model therefore allows us to distinguish the effects of NETs on a vascular endpoint independent of effects related to glucoregulation. Although weight loss and physical activity are the most effective means to prevent or reduce vascular changes induced by obesity^21^, sustained weight loss is difficult to achieve, so treatments designed to prevent the vascular effects of obesity are needed.

**Table 2:** Metabolic parameters of control and DIO mice.

	WT lean	DIO WT	DIO + Cl-amidine	DIO + DNase
Body weight (g)	28.6 ± 0.3	41.8 ± 1.1^†^	39.0 ± 1.5^†^	41.0 ± 1.1^†^
Fasting glucose (mg/dl)	108.4 ± 9.4	173.6 ± 12.3^†^	152.2 ± 17.8^*^	173.3 ± 13.8^†^
Fasting insulin (ng/ml)	0.48 ± 0.04	0.88 ± 0.17^*^	0.63 ± 0.11	0.75 ± 0.16

^*^P < 0.05 compared with WT lean. ^†^P < 0.01 compared with WT lean.

**Figure 1:**
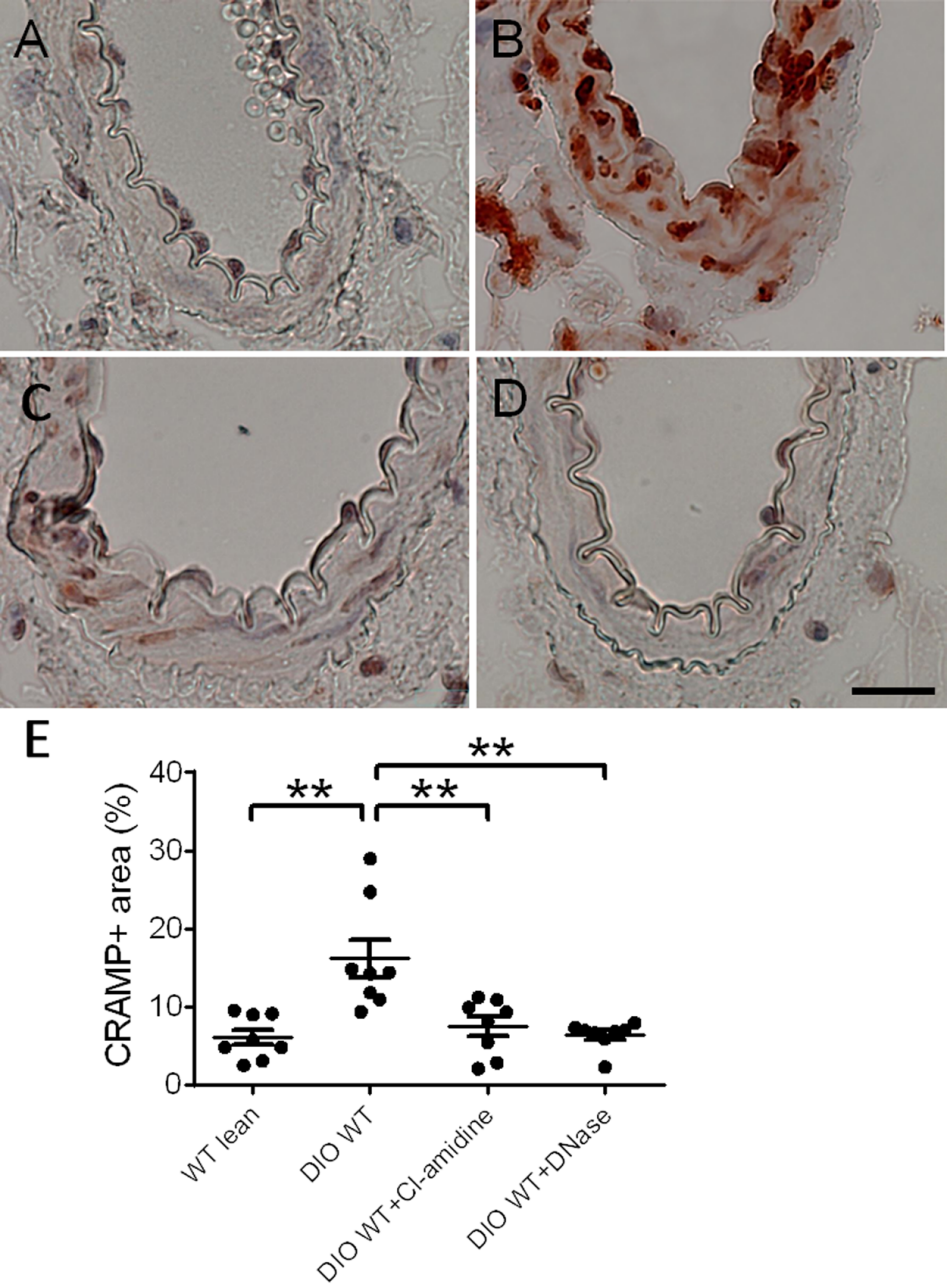
Cathelicidin-related antimicrobial peptide (CRAMP) staining of mesenteric arterioles from WT lean and DIO WT mice with Cl-amidine or DNase treatment (n = 8 mice per group). A, B, C, D: Representative photomicrograph of CRAMP staining in cross sections of mesenteric arterioles from WT lean mice (**A**), DIO WT mice (**B**), DIO WT mice after Cl-amidine treatment (**C**), and DIO WT mice after DNase treatment (**D**). (**E**) Quantification of CRAMP-positive area per unit medial area. *P < 0.05. **P < 0.01. Scale: 20 μm.

To first determine whether obesity affected NET formation in the wall of mesenteric arterioles, we performed immunostaining of cross sections of mesenteric arterioles for CRAMP, which has been used as a surrogate for neutrophils undergoing NET formation in vascular tissue^20–23^. CRAMP immunostaining in mesenteric arterioles was markedly increased in DIO mice compared to control lean mice (Fig. 1E). Following treatment with either 2 weeks of Cl-amidine or 8 days of DNase, CRAMP immunostaining was significantly reduced in mesenteric arterioles of DIO mice compared to vehicle-treated mice (Fig. 1E).

**Figure 2:**
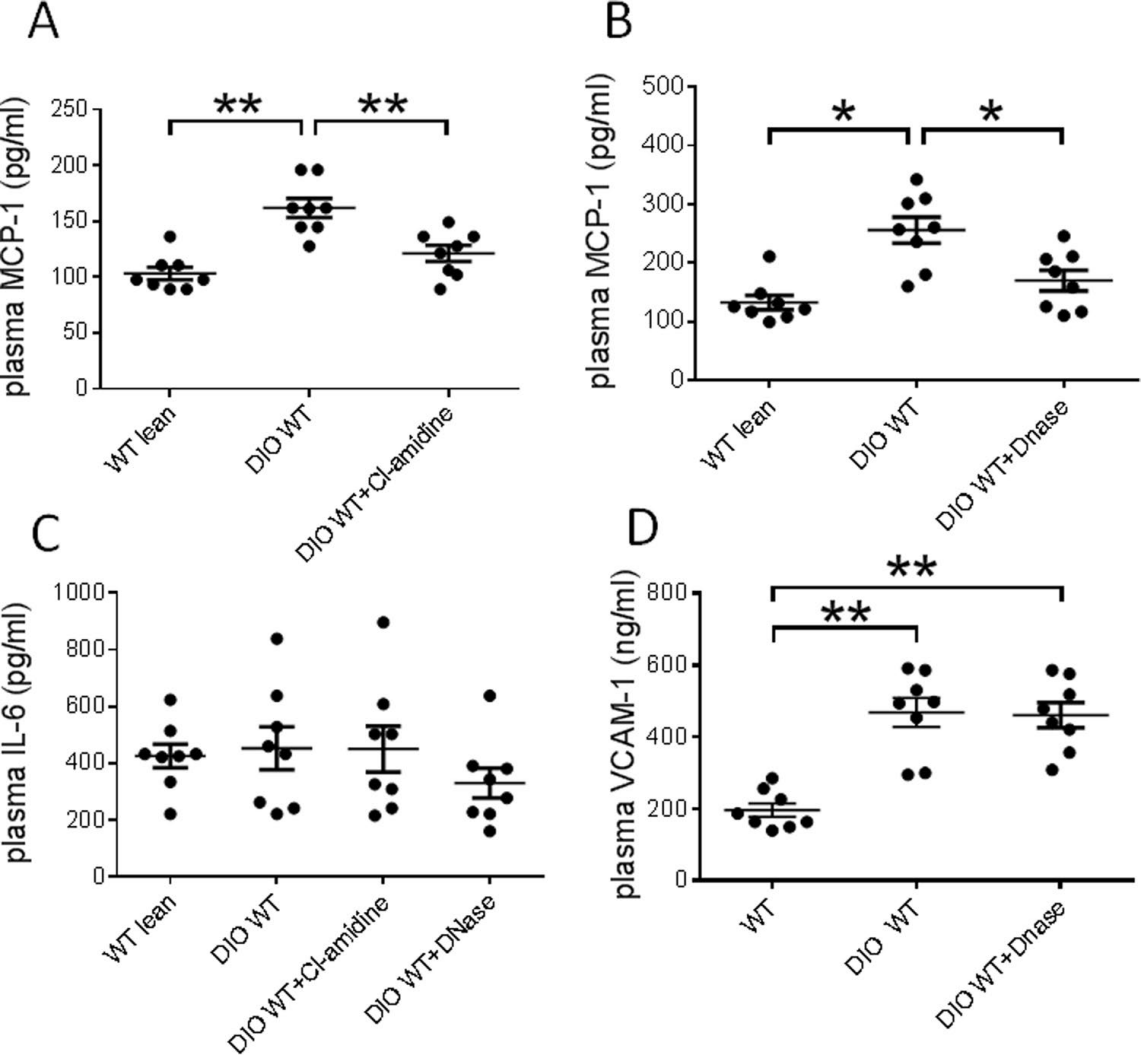
Plasma levels of monocyte chemoattractant protein-1 (MCP-1) and interleukin-6 (IL-6) (n = 8 mice per group). (**A**) Levels of MCP-1in WT lean and DIO WT mice with or without Cl-amidine treatment. (**B**) Levels of MCP-1 in WT lean and DIO WT mice with or without DNase treatment. (**C**) Levels of IL-6 in WT lean and DIO WT mice after Cl-amidine or DNase treatment. (**D**) Levels of VCAM-1 in WT lean and DIO WT mice with or without DNase treatment. *P < 0.05. **P < 0.01.

To determine the effect of DIO on circulating inflammatory markers, the levels of MCP-1, IL-6 and ICAM-1 were measured from lean mice and DIO mice. MCP-1 is a potent monocyte chemotactic factor which known to contribute to disease pathologies stemming from endothelial dysfunction, such as atherosclerosis^24–27^. Circulating concentrations of MCP-1 are higher in obese patients^28^. Consistently, DIO mice were observed to have elevated plasma MCP-1 concentrations relative to mice on normal chow diets (Fig. 2A,B). Interestingly, MCP-1 levels were significantly decreased in DIO mice by either Cl-amidine or DNase treatment (Fig. 2A,B). As MCP-1 expression has been linked to cardiovascular disease progression^24–27^, these observations further support the hypothesis that NET formation contributes to DIO-mediated inflammatory processes.

High concentrations of cell-free DNA have also been shown to correlate with IL-6 levels^29^. Therefore, plasma concentrations of IL-6 were also assayed. However, unlike MCP-1, IL-6 levels were not observed to increase in DIO mice and were not significantly affected by Cl-amidine or Dnase treatment (Fig. 2C). Levels of VCAM-1 were elevated following DIO but not reduced with Dnase treatment (Fig. 2D).

**Figure 3:**
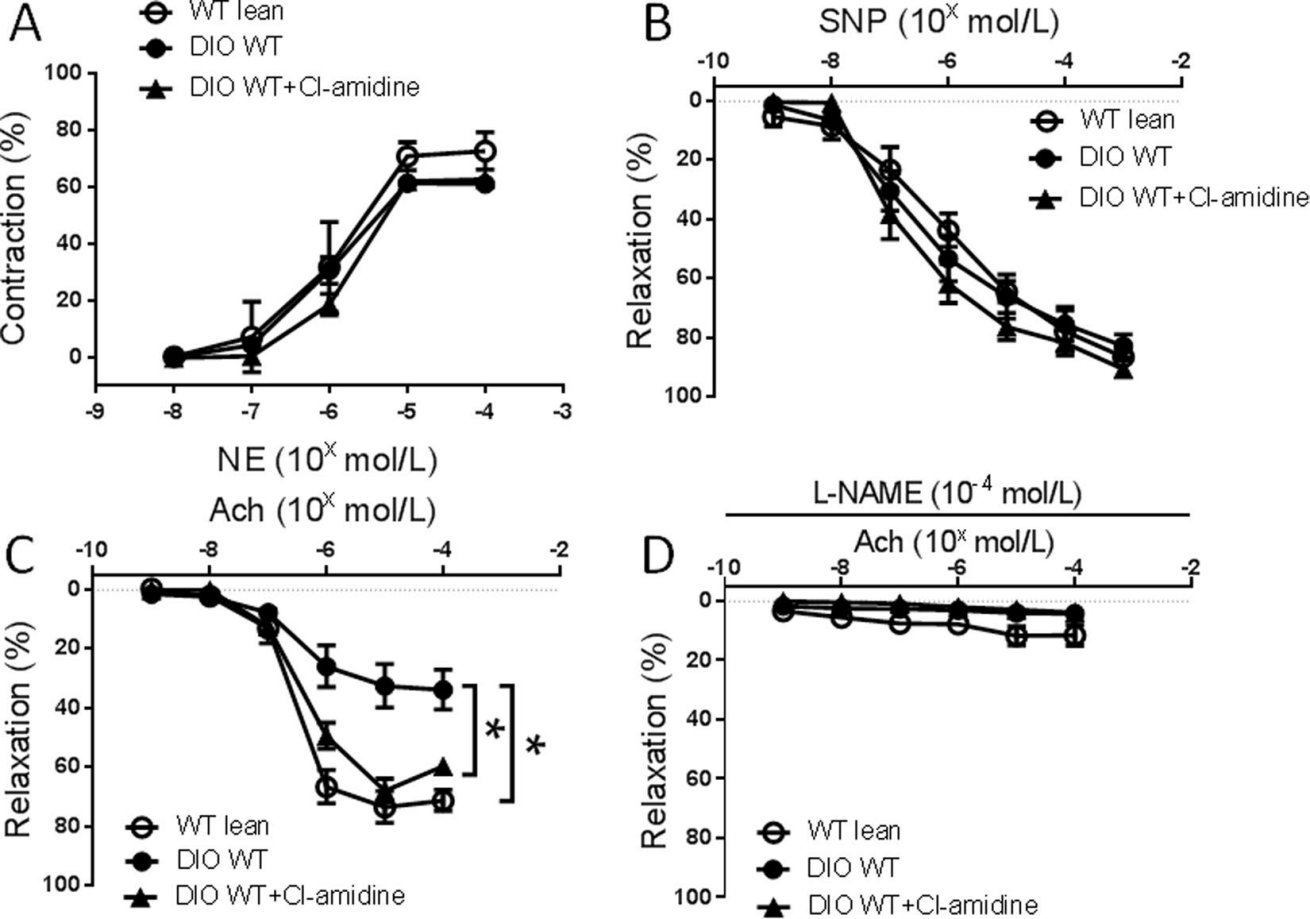
Vasoconstriction and vasorelaxation responses of mesenteric arterioles from control WT lean mice or diet-induced obese (DIO) mice with or without Cl-amidine treatment (n = 8 mice per group). (**A**) Concentration response to norepinephrine (NE). (**B**) Concentration response to sodium nitroprusside (SNP). (**C**) Concentration response to acetylcholine (Ach). (**D**) Concentration response to Ach after preincubation in L-NAME. *P < 0.01.

Endothelial dysfunction is one of the earliest vascular abnormalities related to obesity and the metabolic syndrome, preceding atherosclerosis^30^. To examine the effect of high fat, high sucrose diet on vascular function, pressure myography was performed on WT mice after 10 weeks of diet challenge. NE-induced concentration-dependent contractile responses in mesenteric arteries were similar between WT lean mice and DIO mice (Fig. 3A). Endothelium-independent vasorelaxation responses to SNP were also similar between the groups (Fig. 3B). Endothelial-dependent vasorelaxation was evaluated with Ach. Vasorelaxation responses to Ach were significantly reduced in DIO mice compared to standard chow-fed lean mice (Fig. 3B). To determine the role of NETs in mediating endothelial dysfunction induced by DIO, obese mice were studied after 2 weeks of Cl-amidine treatment. Ach-induced vasorelaxation was significantly improved after Cl-amidine in DIO mice compared to DIO mice without treatment (Fig. 3C). NE-induced vasoconstriction or SNP-induced endothelium-independent vasorelaxation were similar between the groups (Fig. 3A,B). Ach-induced vasorelaxation was inhibited in all groups after preincubation with L-NAME (Fig. 3D).

**Figure 4:**
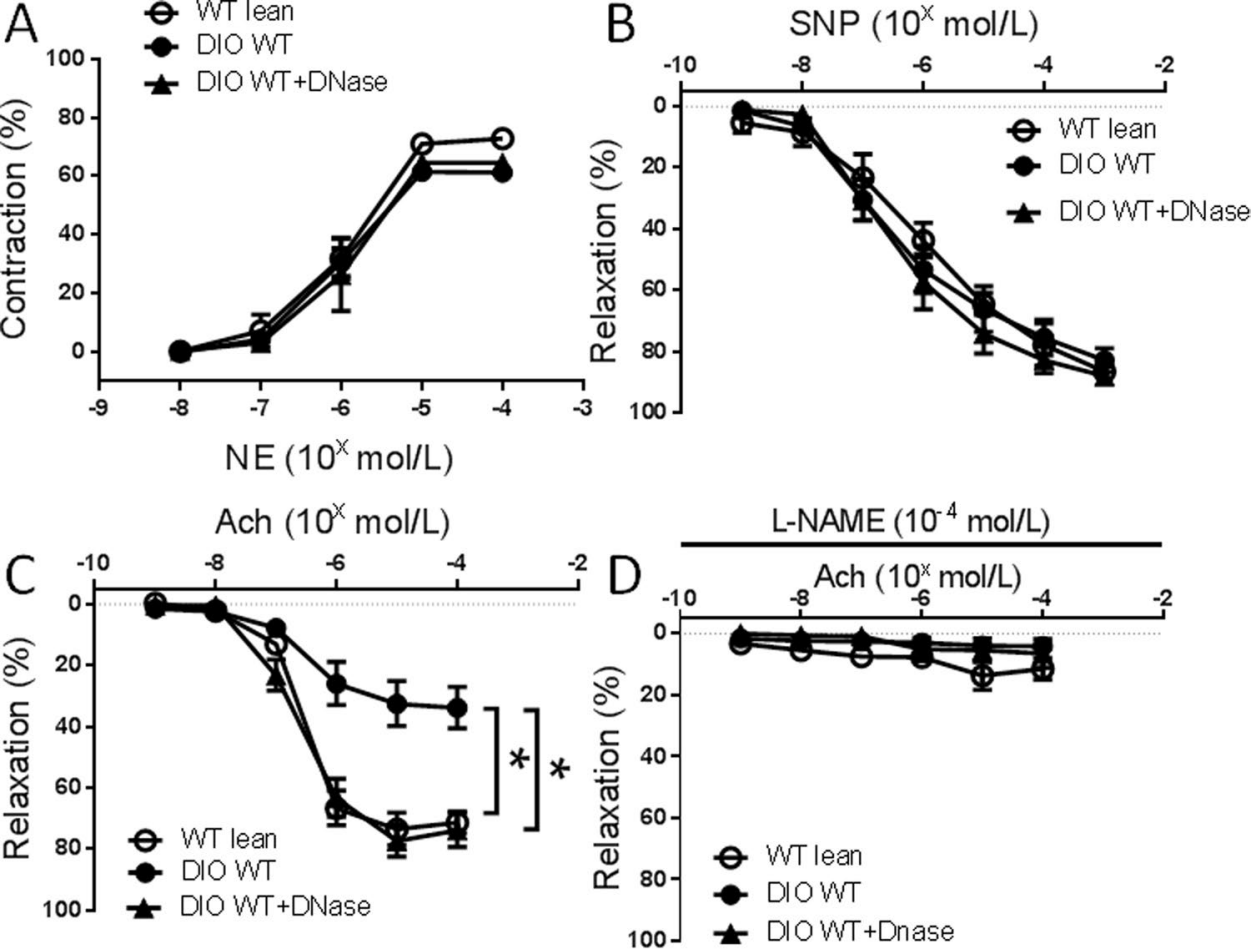
Vasoconstriction and vasorelaxation responses of mesenteric arterioles from control WT lean mice or diet-induced obese (DIO) mice with or without DNase treatment (n = 8 mice per group). (**A**) Concentration response to norepinephrine (NE). (**B**) Concentration response to sodium nitroprusside (SNP). C: Concentration response to acetylcholine (Ach). (**D**) Concentration response to Ach after preincubation in L-NAME. *P < 0.01.

Degradation of NETs with DNase was also used to test the effect of NET formation on DIO-induced endothelial dysfunction. Eight days of treatment with DNase was sufficient to recover the endothelial dysfunction induced by DIO (Fig. 4C). DNase had no effect on NE-induced vasoconstriction and SNP-induced endothelium-independent vasorelaxation between the groups (Fig. 4A,B). Ach-induced vasorelaxation was inhibited in all groups after preincubation with L-NAME (Fig. 4D).

Possible mechanisms by which NET accumulation may affect endothelial function are many and include myeloperoxidase (MPO) present on the DNA extruded from neutrophils during NET formation^31^. MPO can catalyze free radical production, leading to oxidization of endothelial-derived NO^32,33^. Thus, inhibition of NET formation could restore bioavailability of NO to the arteries though not all studies have shown vascular benefits with DNase treatment^34^.

In summary, this study revealed that endothelial function can be recovered after diet-induced endothelial dysfunction by targeting NET formation, in the absence of weight loss or reduction in serum glucose and insulin. This finding establishes NET formation as a driving factor underlying endothelial dysfunction. The agents used to inhibit NET formation in this study, recombinant DNAse and Cl-amidine, have been found to be safe for patients in a Phase I trial^35^ and to have no toxic effects in mouse studies^36^. Therefore, NET inhibition may prove to be a viable method for the treatment of obesity-related endothelial dysfunction and inflammation.
